# Large-scale prediction and analysis of protein sub-mitochondrial localization with DeepMito

**DOI:** 10.1186/s12859-020-03617-z

**Published:** 2020-09-16

**Authors:** Castrense Savojardo, Pier Luigi Martelli, Giacomo Tartari, Rita Casadio

**Affiliations:** 1 Department of Pharmacy and Biotechnology (FaBiT), Biocomputing Group, University of Bologna, Bologna, Italy; 2 Institute of Biomembranes, Bioenergetics and Molecular Biotechnologies (IBIOM), Italian National Research Council (CNR), Bari, Italy

**Keywords:** Submitochondrial localization, Subcellular localization, Mitochondrial protein, Convolutional neural network, Deep learning, Functional annotation

## Abstract

**Background:**

The prediction of protein subcellular localization is a key step of the big effort towards protein functional annotation. Many computational methods exist to identify high-level protein subcellular compartments such as nucleus, cytoplasm or organelles. However, many organelles, like mitochondria, have their own internal compartmentalization. Knowing the precise location of a protein inside mitochondria is crucial for its accurate functional characterization. We recently developed DeepMito, a new method based on a 1-Dimensional Convolutional Neural Network (1D-CNN) architecture outperforming other similar approaches available in literature.

**Results:**

Here, we explore the adoption of DeepMito for the large-scale annotation of four sub-mitochondrial localizations on mitochondrial proteomes of five different species, including human, mouse, fly, yeast and *Arabidopsis thaliana*. A significant fraction of the proteins from these organisms lacked experimental information about sub-mitochondrial localization. We adopted DeepMito to fill the gap, providing complete characterization of protein localization at sub-mitochondrial level for each protein of the five proteomes. Moreover, we identified novel mitochondrial proteins fishing on the set of proteins lacking any subcellular localization annotation using available state-of-the-art subcellular localization predictors. We finally performed additional functional characterization of proteins predicted by DeepMito as localized into the four different sub-mitochondrial compartments using both available experimental and predicted GO terms. All data generated in this study were collected into a database called DeepMitoDB (available at http://busca.biocomp.unibo.it/deepmitodb), providing complete functional characterization of 4307 mitochondrial proteins from the five species.

**Conclusions:**

DeepMitoDB offers a comprehensive view of mitochondrial proteins, including experimental and predicted fine-grain sub-cellular localization and annotated and predicted functional annotations. The database complements other similar resources providing characterization of new proteins. Furthermore, it is also unique in including localization information at the sub-mitochondrial level. For this reason, we believe that DeepMitoDB can be a valuable resource for mitochondrial research.

## Background

Given the unprecedented amount of DNA and protein sequences made available thanks to modern sequencing technologies, a major challenge becomes the ability to make sense of these data. To date, the UniprotKB database [1] contains about 172 million protein sequences, 560 thousand out of which are part of the manually curated Swiss-Prot database. However, given the high costs associated with experimental characterization, only a small fraction of the sequences has experimental functional annotation. In this context, computational tools for automatic functional annotation are of prominent importance in an attempt of filling the gap, as testified by the high number of research groups participating in the Critical Assessment of protein Function Annotation algorithms (CAFA) [2]. Protein subcellular localization (SL) is one of the key aspects defining protein function. Many computational methods have been published in the past, addressing the problem of predicting protein localization starting from protein sequence [3, 4]. Available tools roughly belong to two main categories: (i) approaches that try to detect specific sorting signals such as signal or organelle-targeting peptides [5–11]; (ii) general methods predicting SL using features extracted from the entire protein sequence [12–17]. In both cases, methods mainly predict the localization of proteins into main compartments, such as nucleus, cytoplasm, organelles, ER, plasma membrane and extracellular space. Many cytoplasmic organelles have their own internal structure. In particular, a double membrane comprising an outer and an inner membrane, separated by an intermembrane space, surrounds mitochondria. The inner membrane encapsulates the mitochondrial matrix. Mitochondrial compartments contain different protein types and play different roles in the overall mitochondrial activity. Computational methods, attempting to localize proteins in suborganelle compartments, have been substantially limited so far by the lack of experimental data. Concerning submitochondrial localization, few methods exist, discriminating three main compartments: outer membrane, inner membrane and matrix [18–25]. Recently, we develop DeepMito [26], a novel approach based on convolutional neural networks, able to discriminate all four mitochondrial compartments and outperforming other methods in literature [27].

Here we explore the adoption of DeepMito for performing proteome-wide prediction of sub-mitochondrial localization on representative proteomes of five species including human, mouse, yeast, fly and *Arabidopsis thaliana*. Firstly, we assessed for each proteome the predictive performance of DeepMito on the set of proteins already endowed with manually curated submitochondrial localization. DeepMito reported a very good prediction performance, further confirming its effectiveness in the discrimination of submitochondrial compartments. Secondly, we endowed with submitochondrial localization 1024 proteins already experimentally annotated as mitochondrial but lacking the submitochondrial localization. Furthermore, we identified novel mitochondrial proteins among those lacking any SL annotation using three different SL predictors. The predicted mitochondrial proteins were further analyzed using DeepMito, achieving an additional full characterization of 2530 proteins.

We completed the above analysis with a thorough functional characterization with biological process and molecular function annotations available in UniProtKB and, when necessary, predicted by means of our Bologna Annotation Resource [28].

Overall, we were able to annotate 4307 mitochondrial proteins. We collected the results of our annotation in a new database called DeepMitoDB, accessible at http://busca.biocomp.unibo.it/deepmitodb. DeepMitoDB stands as a comprehensive resource for mitochondrial proteins, collecting together proteome-wide experimental and predicted mitochondrial localization, as well as rich functional annotations for the five model organisms.

## Results

### Available annotations for the five reference proteomes

In Table 1, we report the present status of the annotation of subcellular localization in UniProtKB for the five different proteomes considered. We generated all the data starting from the UniProtKB release of July 2019 (see Methods section for details on the procedure adopted to generate the protein dataset). Overall, we collected some 90,237 proteins for the five organisms. Significant fractions of proteins, even in well-annotated organisms like humans, still lack any SL annotation (20, 28, 21, 76 and 55% of human, mouse, yeast, fly and *Arabidopsis thaliana* proteomes, respectively). Moreover, for many proteins, the annotation is electronically inferred and/or predicted and without experimental evidence.

**Table 1:** The present status of Subcellular Location (SL) annotations of five reference proteomes

	Human	Mouse	Yeast	Fly	Arabidopsis
**# of proteins (UniProtKB reference proteome)**	20,667	22,259	6049	13,796	27,466
**# of proteins with only experimental mitochondrial SL**	350	120	278	32	244
**# of proteins with experimental sub-mitochondrial SL**	263	97	251	12	130
**# of proteins with experimental non-mitochondrial SL**	6178	3561	2208	895	3642
**# of protein without experimental SL**	9772	12,244	2022	2373	8309
**# of proteins lacking SL annotation**	4104	6237	1290	10,484	15,141

The availability of experimental (ECO:000269) annotations for SL varies across the five different species, accounting for 33, 17, 45, 7 and 15% of the proteins from human, mouse, yeast, fly and *Arabidopsis thaliana*, respectively. 5–9% of the proteins is experimentally annotated as being localized into mitochondria in all species but yeast, of which known mitochondrial proteome accounts for 19% of all experimentally annotated proteins. The fractions of mitochondrial proteins for which a more specific sub-mitochondrial SL is experimentally available are 43, 45, 47, 27 and 35% in human, mouse, yeast, fly and *Arabidopsis thaliana*, respectively.

In Table 2, we report a detailed statistics of available SL annotations of the different sub-mitochondrial levels. As a general pattern for all species, inner membrane proteins represent the most abundant class, followed by matrix, outer membrane and intermembrane space proteins. The vast majority of proteins is annotated as localized into a single sub-mitochondrial compartment, while only a small fraction is annotated as having multiple subcellular localizations.

**Table 2:** Compartment-level statistics of experimental (ECO:000269) sub-mitochondrial localizations

Localization	Human	Mouse	Yeast	Fly	Arabidopsis
Outer membrane	61	26	35	3	21
Inner membrane	92	41	106	4	57
Intermembrane space	11	4	37	3	6
Matrix	77	21	54	2	41
***Single localization***	***241***	***92***	***232***	***12***	***125***
***Multi-localized***	***22***	***5***	***19***	***0***	***5***
**Total**	**263**	**97**	**251**	**12**	**130**

### Assessing DeepMito performance on the reference proteomes

We previously described the performance of DeepMito under different conditions and on different datasets [26]. Here, we are interested in evaluating the tool at work on full-proteome analysis. As a first experiment, we assessed DeepMito performance on the five proteomes considered in this work. In particular, we analyzed with DeepMito the subsets proteins with the two following characteristics: i) belonging to the five different species and ii) endowed with single-compartment experimental annotation of SL at the sub-mitochondrial level (evidence code ECO:0000269). As reported in Table 2, this dataset comprises 702 proteins where 241, 92, 232, 12 and 125 are from human, mouse, yeast, fly and *Arabidopsis thaliana*, respectively. We scored the performance of DeepMito by computing Matthews Correlation Coefficients (MCCs) for each sub-mitochondrial compartment assignment (Table 3). Since some species contain only few protein sequences (e.g. fly), we also report aggregated results considering the whole dataset (column Overall in Table 3). As previously highlighted [26], performance of DeepMito is stable across the four different compartments, ranging from 0.73 (intermembrane space and matrix) to 0.81 (outer membrane) MCC values. Interestingly, the 0.73 MCC value for the intermembrane space proteins indicates only a small performance drop for this compartment, which contains the smallest fraction of proteins. The finding indicates the good ability of DeepMito to deal with class imbalance, and this is one of the major advantages of our method over previously released approaches [26, 27].

**Table 3:** Prediction performance (MCC) of DeepMito on the sets of proteins endowed with experimental (ECO:000269) sub-mitochondrial localization

Class/Species	Human	Mouse	Yeast	Fly ^**(a)**^	Arabidopsis	Overall
**Outer membrane**	0.87	0.67	0.75	1.0	0.85	0.81
**Inner membrane**	0.78	0.72	0.78	0.71	0.79	0.77
**Intermembrane space**	0.72	0.56	0.74	0.77	0.86	0.73
**Matrix**	0.72	0.57	0.80	0.67	0.76	0.73

^(a)^Only 12 proteins are included in this dataset

### Proteome-wide annotation of sub-mitochondrial localization

In order to provide complete characterization of protein localization at sub-mitochondrial level we analyzed two sets of proteins with DeepMito:

1. Proteins endowed with experimental mitochondrial localization (ECO:000269) but lacking experimental evidence at sub-mitochondrial level. This dataset will be referred to as MitoExp.
2. The remaining proteins lacking any annotation for SL and/or endowed with non-experimental localization. This dataset will be referred to as MitoPotential

The MitoExp dataset comprises 1024 mitochondrial proteins distributed into 350, 120, 278, 32 and 244 sequences from human, mouse, yeast, fly and *Arabidopsis thaliana*, respectively. We analyzed DeepMito for sake of increasing the annotation granularity of proteins in sub-mitochondrial compartments.

The MitoPotential dataset includes 71,976 proteins distributed into 18,481, 12,857, 3312, 13,876 and 23,450 sequences from human, mouse, yeast, fly and *Arabidopsis thaliana*, respectively. Sequences in the MitoPotential dataset are preprocessed for the identification of putative mitochondrial proteins. Specifically, we performed in-silico prediction of SL using three different state-of-the-art tools: BUSCA [14], MitoFates [10] and TargetP-2.0 [8]. A query protein is considered as localized into mitochondria if two out of three methods agreed on its mitochondrial localization. By this, we identified 2530 novel mitochondrial proteins on which we performed DeepMito analysis.

**Fig. 1:**
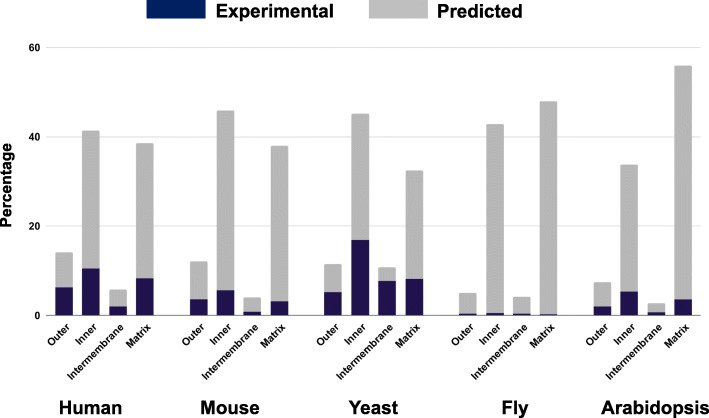
Distributions of combined experimental and predicted sub-mitochondrial compartments for the five species considered in this study

We merged the results of our experiments with the remaining 753 proteins with available experimental annotations for the sub-mitochondrial localization (see Table 1). This allowed obtaining the submitochondrial annotation of the proteome of the five species considered, overall characterizing 4307 protein sequences. Figure 1 and Table 4 show the distributions of sub-mitochondrial compartments (predicted and true) in the proteomes.

**Table 4:** Summary of sub-mitochondrial localizations (annotated and predicted) of the 4307 mitochondrial proteins

Class/Species	Human	Mouse	Yeast	Fly	Arabidopsis	Overall
**Outer membrane**	151	93	82	34	85	445
**Inner membrane**	441	354	321	288	386	1790
**Intermembrane space**	62	31	77	28	31	229
**Matrix**	412	293	231	323	638	1897
**Total**	1066	771	711	673	1140	4361 ^(a)^

^(a)^The total count is greater than 4307 because of the presence of multi-localizing proteins

For all species, inner membrane and matrix proteins are the most abundant, whereas outer membrane and intermembrane space proteins account for smaller fractions (both experimental and predicted, with the only exception of yeast in which we observe a high number of experimentally annotated intermembrane proteins). In human and mouse, the overall fractions of inner membrane and matrix proteins are quite similar. In yeast we observe a higher concentration of inner membrane proteins (both considering experimental and predicted distributions) whereas in fly and *Arabidopsis thaliana* the overall percentages of matrix proteins are about 20% more abundant than the inner membrane proteins. These differences may be due to a combined effect including the natural differences between organisms and/or the different amount of experimental evidence observed (as already highlighted in Table 1) that hampers the possibility of building balanced training datasets and leads to uneven distributions of prediction errors between different organisms. When input information is low, performance indeed can be affected by higher instability. In spite of this, data are important for driving possible future experimental validation.

### Functional characterization of proteins residing in different mitochondrial compartments

In order to provide complete functional characterization of the 4307 mitochondrial proteins from the five organisms, we retrieved from the UniProtKB Gene Ontology Annotation (GOA) database (https://www.ebi.ac.uk/GOA/, release 2019_10), available GO annotations on Biological Process (BP) and Molecular Function (MF) aspects. We retrieved All GO annotations, also including those having IEA (Inferred by Electronic Annotation) evidence code. Proteins lacking GO annotations (in one or both aspects) were annotated using our Bologna Annotation Resource (BAR3.0), which allows to transfer statistically validated functional annotations from precomputed clusters including more than 32 million UniProtKB sequences [28]. This allowed to extend GO-BP and GO-MF annotations to 3668 (85%) and 3492 (81%) proteins, respectively. Table 5 summarizes the number of annotated proteins and terms of the two GO subontologies, also reporting for each one how many terms are from GOA with manual curation, from GOA with automatic annotation or assigned with BAR3.0. Multiple identical annotations with different evidence codes were collapsed into a single entry retaining the one with the highest quality evidence.

**Table 5:** Number of proteins annotated with GO-BP and GO-MF in the five proteomes

	GO term	
	BP	MF
**# annotated proteins**	3668	3492
**# annotations (GOA manually reviewed)** ^**(a)**^	15,984	10,346
**# annotations (GOA automatic and unreviewed)** ^**(b)**^	3439	4527
**# annotations (BAR3)** ^**(c)**^	11,053	5433
**Total annotations (# different terms)**	30,476 (5381)	20,306 (2133)

^(a)^Evidence codes: all except IEA

^(b)^IEA evidence code

^(c)^Terms uniquely annotated by BAR3.0

Overall, 5381 and 2133 different GO-BP and GO-MF terms annotate the 3668 and 3492 mitochondrial proteins, respectively. These correspond to 30,476 and 20,306 associations of GO-BP and GO-MF, respectively. About one third of the GO-BP and one fourth of the GO-MF associations derive from the BAR3.0 platform (Table 5).

Overall, different BP and MF terms annotate proteins in the four compartments. Typical biological processes characterizing specific compartments are: cell death (GO:0008219), observed in the outer membrane; oxidative phosphorylation (GO:0006119) and respiratory electron transport chain (GO:0022904) observed in the inner membrane; phospholipid transport (GO:0015914) and protein import into mitochondrial intermembrane space (GO:0045041) observed in the intermembrane space; and pyruvate metabolic process (GO:0006090) observed in the matrix.

### The DeepMitoDB

We collected all data produced in this study into a database called DeepMitoDB. The database is available at http://busca.biocomp.unibo.it/deepmitodb. DeepMitoDB represents a comprehensive resource for researchers interested in studying mitochondrial proteins. Its major characteristics is to combine proteome-wide experimental data with predicted annotation of subcellular localization at submitochondrial level and complementary functional characterization in terms of biological processes and molecular functions. We extracted this rich functional characterization from available annotations in GOA and complemented with similarity-based annotations carried-out with our BAR 3.0 platform. DeepMitoDB allows users to search for proteins by organisms, mitochondrial compartment, biological process or molecular function and to quickly retrieve and download results in different formats, including JSON and CSV.

## Discussion

### Comparing DeepMitoDB with similar databases

Different existing databases, such as the Integrated Mitochondrial Protein Index (IMPI, available through MitoMiner4.0) [29], MitoCarta [30] and the Human Protein Atlas – Subcellular Localization (HPA-SL) [31], collect mitochondrial localization and related data. None of the databases, at the latest releases, provides any information about sub-mitochondrial localization. In this respect, to the best of our knowledge, DeepMitoDB is the first database providing integrated experimental and predicted evidence for protein localization within mitochondria.

**Fig. 2:**
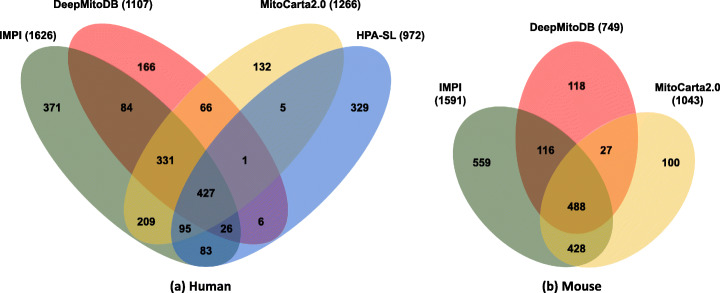
Comparison of data contained in different databases of human (**a**) and mouse (**b**) mitochondrial genes/proteins. In parentheses the number of unique Ensembl gene ID contained in each database

Here we compare DeepMitoDB with IMPI, MitoCarta2.0 and HPA-SL. Since the various databases differ in the set of represented species, we restrict the comparison on human (available in all databases) and mouse (available in all databases but HPA-SL) species. Moreover, since DeepMitoDB is protein-centric while others are gene-centric, we mapped all the data entries in all databases onto stable Ensembl Gene identifiers. Then, we computed the overlap between the databases. Figure 2 shows results for human (a) and mouse (b) genes. Although different databases share significant fractions of genes, each lists a different number of mitochondrial genes (reported in parentheses aside each DB name as the number of unique Ensembl gene ID represented). The differences are likely due to different predictive strategies adopted to identify putative mitochondrial genes and/or the integration of different types of experimental evidence.

DeepMitoDB complements other resources with 166 and 118 human and mouse genes/proteins, respectively. Interestingly, the mitochondrial localization of 84 (51%) and 81 (69%) of the above human and mouse proteins, respectively, was established with the SL prediction methods detailed above.

### DeepMitoDB use case

To show a possible use case of the database, we analyzed human proteins that are uniquely present in DeepMitoDB (166 proteins, as reported in Fig. 2) and we describe here a case demonstrating how our database, integrating experimental and predicted localizations, can be useful for anticipating and refining experimental SL information available.

Specifically, the human Nocturnin protein (UniProtKB accession: Q9UK39), a phosphatase which catalyzes the conversion of NADP^+^ to NAD^+^ and of NADPH to NADH, is involved in the control of circadian clock [32]. The protein was not annotated as localized into mitochondria in the July 2019 UniProtKB release (i.e. the release we used to build our database): for this reason, the protein was included in the MitoPotential dataset and processed by SL predictors. The protein was hence identified as mitochondrial and predicted by DeepMito to be localized into the mitochondrial matrix compartment. Interestingly, experimental mitochondrial localization of this protein was assessed in 2019 [32] and included into a subsequent UniProtKB release (Aug, 2019). In other words, this example shows how a SL database including predicted data can provide SL annotation before experiments are available. Moreover, our database further complements experimental data providing predicted sub-mitochondrial localization, which is not yet available for the specific protein.

## Conclusions

In this work, we performed a large-scale, proteome-wide analysis for annotating mitochondrial and sub-mitochondrial localization of proteins from five well-studied reference proteomes: human, mouse, yeast, fly and *Arabidopsis thaliana*. A significant fraction of the proteins from these organisms still lack functional and sub-cellular localization annotations. We contributed to fill this gap by providing proteome-wide annotations of protein localization at sub-mitochondrial level obtained with our recently developed predictor DeepMito.

Firstly, we scored DeepMito on the set of proteins already characterized at the sub-mitochondrial level: in this experiment, we further confirmed state-of-the-art performance of our tool in a large-scale assessment.

Secondly, the tool was applied to proteins already known to be mitochondrial but lacking sub-mitochondrial localization annotations as well as to new mitochondrial as predicted by the BUSCA webserver. Overall, 4307 proteins were characterized in the five organisms. Information about subcellular localization was then complemented with manually curated and predicted functional annotations from the biological process and molecular function GO ontologies. The data were all collected and are now available through a database called DeepMitoDB accessible at http://busca.biocomp.unibo.it/deepmitodb. The database provides a comprehensive view of mitochondrial proteins, including manually curated and predicted fine-grain sub-cellular localization and annotated and predicted functional annotations. Other resources dedicated to mitochondrial proteins also account for localization data [29–31]. As a complement, DeepMitoDB provides characterization of new proteins. Furthermore, it is also unique in including localization information at the sub-mitochondrial level. For this reason, we believe that DeepMitoDB can be a valuable resource for mitochondrial research.

## Methods

### Datasets

We extracted from UniProtKB release July 2019 the reference proteomes of five different organisms: human, mouse, yeast, fly and *Arabidopsis thaliana*. In particular, from the UniProtKB Proteomes Portal (https://www.uniprot.org/proteomes/) we downloaded FASTA sequences corresponding to reference proteomes and containing one sequence per gene.

Annotations of subcellular localizations were obtained from the corresponding field of the UniProtKB entry and considering as experimental evidence only (ECO:0000269).

Available Gene Ontology (GO) annotations were extracted from the UniProtKB Gene Ontology Annotation (GOA) database (https://www.ebi.ac.uk/GOA/), release October 2019.

### The DeepMito predictor

DeepMito [26] is a recently released predictor of protein submitochondrial localization. DeepMito is one of the few methods available (e.g. SubMitoPred [27]) that is able to discriminate four different mitochondrial compartments, namely the two membranes (inner and outer), the intermembrane space e the matrix.

Here, we provide a general overview of the method. Additional details on the DeepMito architecture as well as on the training procedure can be found on the reference paper [26].

**Fig. 3:**
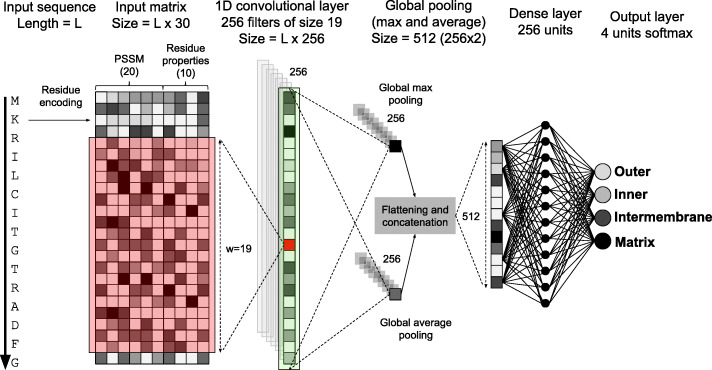
The DeepMito convolutional network architecture

The method relies on the convolutional neural network architecture shown in Fig. 3.

Input proteins are encoded using two different descriptors:

1. Evolutionary information, in the form of Position Specific Scoring Matrices (PSSMs) as obtained running PSI-BLAST [33] (three iterations, e-value 0.001) against the Uniref90 database (release March, 2018). This encodes each residue using 20 different values.
2. Residue physical–chemical attributes, in particular using the 10 different numerical values introduced by Kidera and coworkers [34]. These values were obtained by means of a multivariate statistical analysis starting from a set of 188 different properties.

Overall, a protein is encoded by a Lx30 matrix, where L is the length of the protein. This input is processed using a convolutional neural network including the following layers:

1. A 1D convolutional layer comprising 256 filters each of size 19. Each filter scans the encoded input sequence for motifs in the feature space, producing an output of length L. Overall, the layer maps the input protein into a feature map of size Lx256.
2. Two global pooling layers are applied in parallel computing, respectively, the maximum and the average values of each filter scan over the entire sequence length. The outputs of these layers, each of size 256, are then combined together and flattened into a vector of 512 values.
3. An output fully connected network comprising 256 hidden units mapping the combined pooling output into the four output units corresponding to the four mitochondrial compartments. The softmax activation function is applied at the layer output (obtaining a probability output for each class) and the predicted compartment is the one having the highest probability.

DeepMito was trained and tested in cross-validation on a dataset comprising 424 high-quality, non-redundant proteins extracted from UniProtKB/SwissProt (release February 2018) and experimentally annotated as being localized into one of the four mitochondrial compartments. In all comparative experiments, DeepMito outperformed other approaches, showing, in particular, a very high robustness with respect to class imbalance [26].

### Other prediction methods

#### Prediction of subcellular localization

The Bologna Unified Subcellular Component Annotator (BUSCA, http://busca.biocomp.unibo.it) [14] is a web-server integrating several different tools for predicting protein subcellular localization of both globular and membrane proteins as well as methods for identifying localization-related features such as signal and transit peptides, GPI-anchors and transmembrane regions. These tools are organized into a decision graph processing the outputs of the different tools. Overall, BUSCA an input protein can be classified by BUSCA into one of many cellular compartments. The number of discriminated localizations depends on the taxonomic origin of the input protein: BUSCA supports discrimination of up to 9 localization for animal and fungal protein, 16 compartments for plants (including also chloroplast and sub-chloroplast localizations), 4 for Gram- and 3 for Gram+ bacteria.

Here, BUSCA was adopted for the discovery of novel mitochondrial protein. Given a set of input proteins, BUSCA was then used to discriminate mitochondrial from non-mitochondrial proteins. Simply, all proteins predicted as localized into a compartment different from “mitochondrion” were classified as non-mitochondrial.

Two additional methods were used to complement and verify BUSCA predictions. In particular, the latest version of TargetP (v2.0) [8] (http://www.cbs.dtu.dk/services/TargetP/) was downloaded and ran locally. TargetP-2.0 is based on deep learning methods and predicts mitochondrial localization by recognition of the targeting pre-sequence. Finally, MitoFates [10] (http://mitf.cbrc.jp/MitoFates/cgi-bin/top.cgi), another machine learning-based method recognizing mitochondrial-targeting peptides, was also employed. In this case, proteins were directly uploaded and analyzed using the predictor web server (which allows analyzing up to 2000 proteins per job).

The output of the three above tools were combined by means of a simple majority rule: proteins were considered as localized into mitochondria if at least two out three tools predict the protein as localized into the organelle.

#### Functional annotation

The Bologna Annotation Resource (BAR3.0, https://bar.biocomp.unibo.it/bar3/) [28] is a server for the annotation of protein function. The method implemented by BAR3.0 relies on a comparative large-scale analysis of the entire UniProtKB database. In particular, all sequences included in UniProtKB (considering both SwissProt and TrEMBL) were pairwise aligned using BLAST. From these alignments, a set of clusters was computed grouping all sequences sharing more than 40% sequence identity on at least 90% of the alignment into the same cluster. For all proteins within a cluster, all available experimental annotations, including GO terms and Pfam domains, were extracted. Terms and domains that are over-represented (as obtained from a Bonferroni-corrected Fisher test and setting a 1% significance level) can be transferred on all remaining sequences in the clusters. New sequences enter a cluster via BLAST alignment against the entire database of sequences (the cluster assigned is the one in which is placed the best hit) and inherit all annotations from it. Currently, BAR3.0 contains 28,869,663 sequences (from UniProtKB release May 2015) grouped in 1,361,773 clusters, along with 3,399,026 isolated sequences, called singleton.

Here we used BAR3 to assign GO terms in the biological process and molecular function aspects annotations to complement functional annotation of mitochondrial proteins. In particular, BAR3.0 predictions were merged together with available annotations. In case of multiple annotations of the same GO term, we retained the one having the highest quality evidence code.

## Data Availability

The DeepMitoDB database is accessible at http://busca.biocomp.unibo.it/deepmitodb.

## Abbreviations

1D: One-dimensional
SL: Subcellular localization
MCC: Matthews Correlation Coefficient
PSSM: Position-Specific Scoring Matrix
SI: Sequence identity
GO: Gene ontology
BP: Biological process
MF: Molecular function
GOA: Gene ontology annotation
IEA: Inferred from electronic annotation
IMPI: Integrated Mitochondrial Protein Index
HPA-SL: Human Protein Atlas – Subcellular Localization
